# In situ observation of melt pool evolution in ultrasonic vibration-assisted directed energy deposition

**DOI:** 10.1038/s41598-023-44108-4

**Published:** 2023-10-17

**Authors:** Salma A. El-Azab, Cheng Zhang, Sen Jiang, Aleksandra L. Vyatskikh, Lorenzo Valdevit, Enrique J. Lavernia, Julie M. Schoenung

**Affiliations:** Department of Materials Science and Engineering, University of California, 716 Engineering Tower, Irvine, CA 92697 USA

**Keywords:** Design, synthesis and processing, Characterization and analytical techniques

## Abstract

The presence of defects, such as pores, in materials processed using additive manufacturing represents a challenge during the manufacturing of many engineering components. Recently, ultrasonic vibration-assisted (UV-A) directed energy deposition (DED) has been shown to reduce porosity, promote grain refinement, and enhance mechanical performance in metal components. Whereas it is evident that the formation of such microstructural features is affected by the melt pool behavior, the specific mechanisms by which ultrasonic vibration (UV) influences the melt pool remain elusive. In the present investigation, UV was applied in situ to DED of 316L stainless steel single tracks and bulk parts. For the first time, high-speed video imaging and thermal imaging were implemented in situ to quantitatively correlate the application of UV to melt pool evolution in DED. Extensive imaging data were coupled with in-depth microstructural characterization to develop a statistically robust dataset describing the observed phenomena. Our findings show that UV increases the melt pool peak temperature and dimensions, while improving the wettability of injected particles with the melt pool surface and reducing particle residence time. Near the substrate, we observe that UV results in a 92% decrease in porosity, and a 54% decrease in dendritic arm spacing. The effect of UV on the melt pool is caused by the combined mechanisms of acoustic cavitation, ultrasound absorption, and acoustic streaming. Through in situ imaging we demonstrate quantitatively that these phenomena, acting simultaneously, effectively diminish with increasing build height and size due to acoustic attenuation, consequently decreasing the positive effect of implementing UV-A DED. Thus, this research provides valuable insight into the value of in situ imaging, as well as the effects of UV on DED melt pool dynamics, the stochastic interactions between the melt pool and incoming powder particles, and the limitations of build geometry on the UV-A DED technique.

## Introduction

In casting and other conventional metallurgy processes, dynamic solidification has been utilized to suppress formation of defects and promote nucleation of finer crystals, leading to enhanced mechanical behavior^1^. Dynamic solidification involves forced motion of molten metal ahead of the solidification front, through means such as vibration and electromechanical stirring. Such techniques have been used in metallurgy as early as the 1800s, notably by renowned metallurgist Dmitry Chernov, who reported that vigorously shaking a mold of solidifying steel resulted in a fine crystal structure^1^. Continuing this trend, in recent decades, ultrasonic vibration (UV) has been widely used to assist in casting and welding of metals^2–8^. Ultrasonic waves propagate through solid, liquid, and gas media at frequencies of at least 20 kHz^9^. Due to the nonlinear interactions of ultrasonic waves with molten metal, UV-assisted (UV-A) processing of metals typically leads to fewer defects and finer microstructures, resulting in enhanced mechanical properties^2–8^.

Over the last several years, metal additive manufacturing (AM) has gained popularity as a novel molten metal processing technique, with the development of several laser-based techniques such as directed energy deposition (DED)^10,11^. In DED, a laser creates a melt pool on the surface of a metal substrate, into which metal powders are delivered through a stream of inert gas. The laser incrementally moves up in the z-direction, and either the substrate or the laser raster in the x–y plane. In this fashion, parts are deposited layer-by-layer based on a computer-aided design (CAD) model. DED offers several advantages and capabilities over other conventional and AM techniques, like rapid production time, product repair and performance enhancement, and production of multi-material parts^10,11^.

Analysis of structure–property relationships and optimization of deposition parameters have been carried out for several engineering alloys fabricated via DED, such as Ti-6Al-4V^12,13^, AISI 316L^14,15^ and 304L^16,17^ austenitic stainless steels, AlSi10Mg^18,19^, and nickel-based superalloys such as Inconel 718^20,21^ and Inconel 625^22–24^. These alloys are commonly found in components used in the energy, automotive, and aerospace industries. AISI 316L stainless steel, in particular, is widely used in DED due to its high corrosion resistance and ductility, and low susceptibility to the formation of chromium-rich carbide phases^25–27^. However, DED-fabricated parts are prone to the formation of defects, such as porosity and hot cracking, and irregular microstructures^28–31^. Additionally, due to the presence of extreme temperature gradients within the melt pool, and the stochastic nature of the interactions between the melt pool, laser beam, and powder, DED-fabricated components have a propensity for the formation of and consequential deformation by residual stresses^32,33^. Therefore, an understanding of melt pool dynamics is critical to improve the performance of DED-fabricated parts.

Recently, several studies have confirmed the feasibility of UV-A DED. The microstructures, defects, and mechanical properties of several alloys deposited with UV-A DED have been characterized. For example, Cong and Ning observed an increase in melt pool dimensions, yield strength, ultimate tensile strength (UTS), microhardness, and ductility, and a decrease in porosity in UV-A DED AISI 630 stainless steel single track thin walls^34^. Ning et al. studied the effects of UV on the phase composition, defects, mechanical properties, and melt pool evolution of UV-A DED Inconel 718 parts. They reported a reduction of Laves phases, which are detrimental to the mechanical properties of the Inconel 718 alloy^35^. Additionally, Ning et al. reported a decrease in porosity, and an increase in yield strength, ductility, UTS, and microhardness for Inconel 718^36^. By using an infrared (IR) camera to analyze the melt pool in situ during the deposition of 4-layer components, Ning et al. demonstrated that UV increases the melt pool temperature and dimensions, and that these changes are amplified at higher frequencies^37^. Todaro et al. reported a transition from columnar grains to fine, equiaxed crystals, and an increase in yield strength and tensile strength for UV-A DED-fabricated Ti-6Al-4V bulk parts^38^. Todaro et al*.* also reported a transition from columnar grains to fine, equiaxed crystals of random orientation and a higher density of grains despite a decrease in cooling rate in UV-A DED-fabricated 316L stainless steel parts^39^. These studies show that UV-A DED results in finer microstructures, fewer defects, and improved mechanical properties. This is consistent with what is typically seen in conventional UV-A metallurgy processes, and is promising for the future of metal AM. However, little is understood about how UV affects melt pool evolution particularly as the build dimensions increase, which is essential for scaling up the use of UV-A DED to produce components with large or complex geometries.

The current investigation aims to develop an understanding of how the melt pool temperature, geometry, and interactions with powder are impacted during UV-A DED, and more particularly, how these effects evolve with increasing build size. In this study, 316L stainless steel single tracks and cubes were deposited with and without UV by a proprietary DED technology called Laser Engineered Net Shaping® (LENS®). For the first time, high-speed and thermal imaging were used in situ to visualize the melt pool surface during UV-A LENS® deposition to track UV effects in large builds. Moreover, these techniques provide an extensive and quantitative dataset to measure key melt pool characteristics and thereby correlate them with observed effects of UV on microstructure and build quality. Post-deposition characterization of defects and microstructures in as-built samples was carried out with optical microscopy, scanning electron microscopy (SEM), and electron backscatter diffraction (EBSD). We demonstrate a statistically significant correlation between the application of UV and peak melt pool temperature, melt pool size, and particle residence time. Effects on peak melt pool temperature and melt pool size diminish with increasing build size. We further consider the physical mechanisms by which the UV interacts with the melt pool and the build to provide an explanation of the observed behavior. Overall, this work highlights the power of in situ imaging to quantitatively characterize the UV-A DED process, as well as the limitations that must be considered for future development of UV-A DED when applied to large and/or complex parts.

## Results

**Figure 1:**
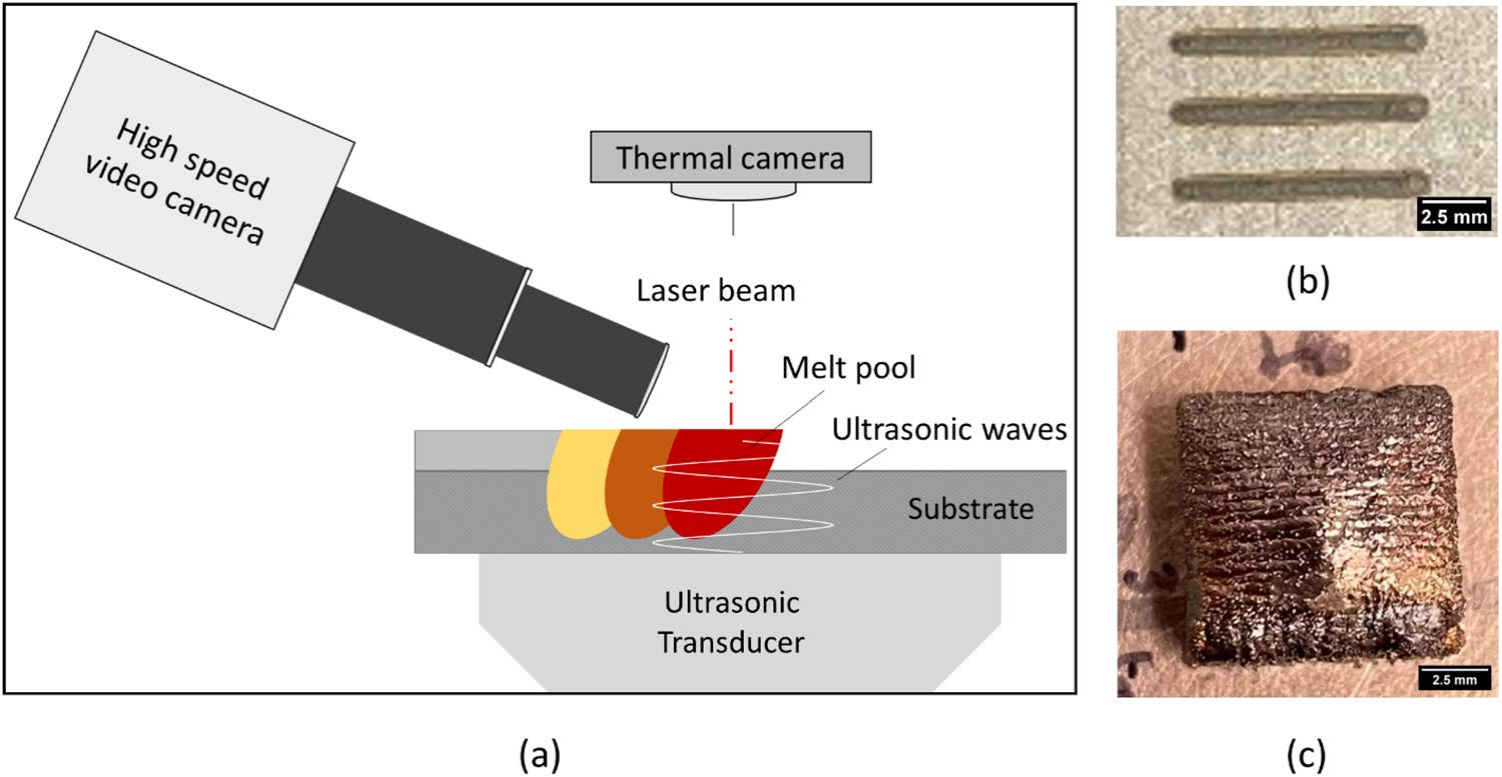
Experimental overview showing: (**a**) The experimental set up (not to scale). The ultrasonic transducer is coupled to the bottom of a 316L stainless steel substrate, sending vibration up into the melt pool during deposition. A high-speed video camera films from the side, and a thermal camera films from above; (**b**) As-deposited single tracks; and (**c**) As-deposited cubic sample.

A piezoelectric ultrasonic transducer was coupled to the bottom-center of a 316L stainless steel substrate. Single-weld (no powder flowing condition), single-track (powder flowing condition), and 40-layer cubic 316L stainless steel samples were deposited using DED with and without UV. A thermal imaging camera and a high-speed video camera were used in situ, as shown in Fig. 1, to image the evolution in melt pool temperature and geometry, and the interactions between the melt pool and injected powder particles, respectively, allowing for the collection of detailed, quantitative data on peak melt pool temperature, melt pool geometry and size, and particle residence time for both deposition conditions—with and without UV. Based on these data and additional post-deposition microstructural characterization on grain size and morphology, melt pool depth, dendrite arm spacing, and porosity, the mechanisms governing UV interactions with the melt pool are described.

### Thermal imaging results

**Figure 2:**
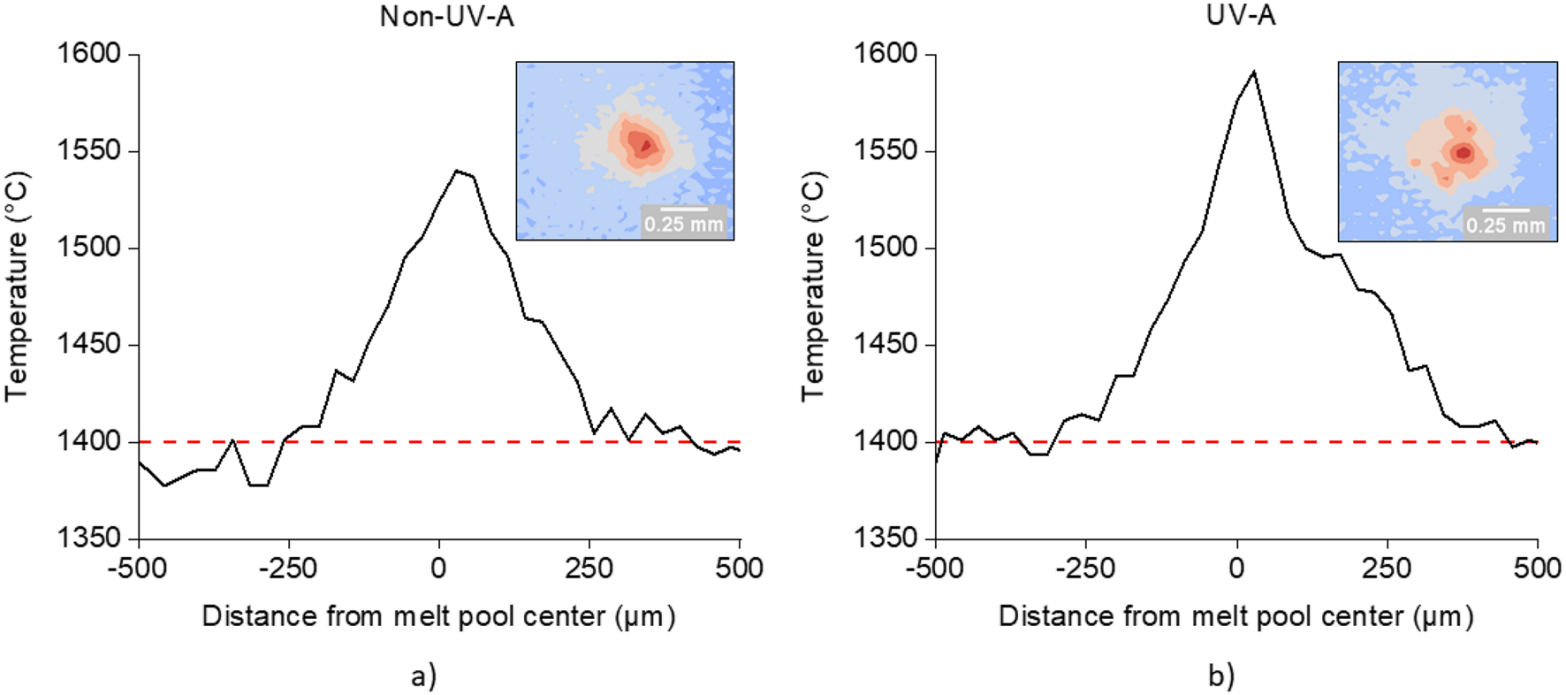
Representative single-track temperature profiles derived from thermal imaging camera contour plots: (**a**) non-UV-A condition, with a narrower, shorter profile, and (**b**) UV-A condition, with a wider, taller profile. The red, dashed lines denote the melting temperature of 316L stainless steel, approximately 1400 °C^25^. The insets show the corresponding thermal contour plot of the melt pools as determined by thermal imaging.

To understand how UV effects melt pool temperature, a thermal camera was used to capture spatial maps of temperature across the melt pool surface. Representative melt pool temperature profiles and thermal contour plots, derived from a single frame captured by the thermal imaging camera, for both non-UV-A and UV-A single tracks (‘powder-flowing’ condition) are shown in Fig. 2. The temperature profile for the UV-A single track is wider and taller than the non-UV-A single track, indicating a larger melt pool with a higher peak temperature when UV is applied during DED.

Average maximum melt pool temperature and average melt pool surface area were calculated from approximately 700 frames for both non-UV-A and UV-A single tracks. For the non-UV-A single track melt pools, the average maximum temperature was 1550 ± 50 °C, and the average surface area was 0.02 ± 0.02 mm^2^ (uncertainty values expressed here and elsewhere in this article are standard deviation values). For the UV-A single track melt pools, the average maximum temperature was 1640 ± 90 °C, and the average surface area was 0.05 ± 0.04 mm^2^.

**Figure 3:**
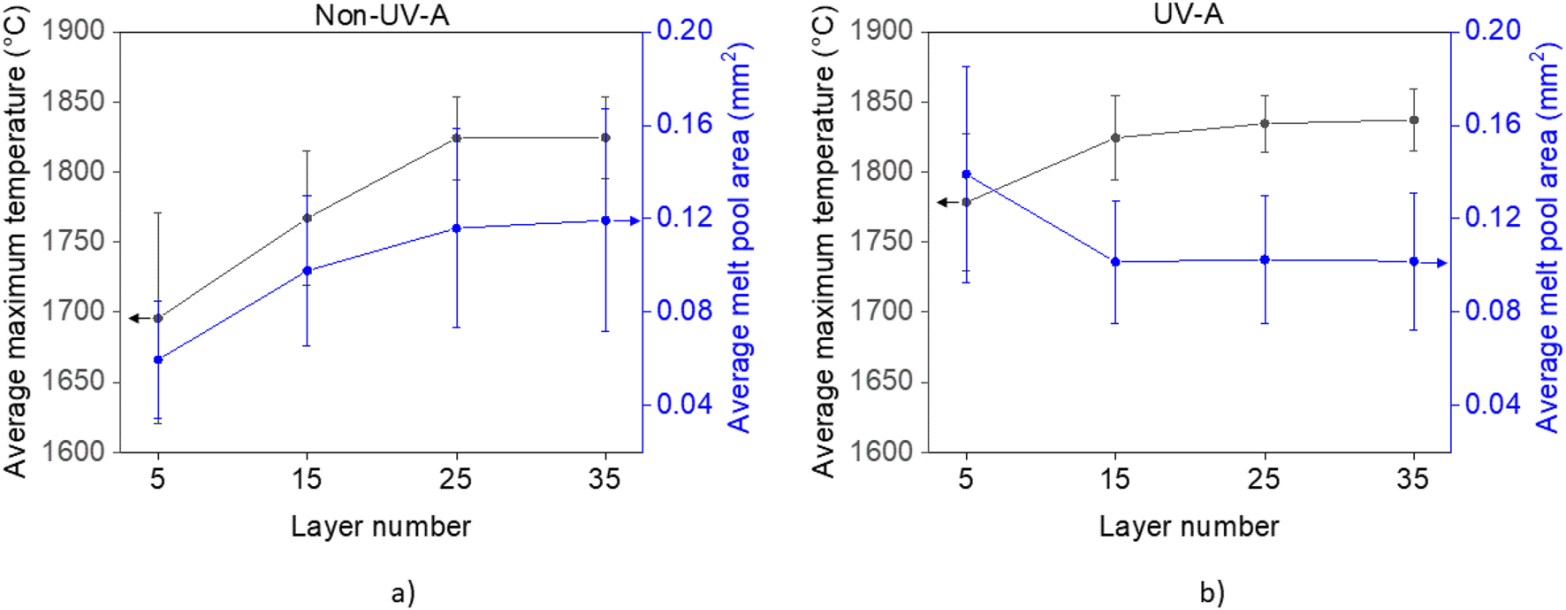
Melt pool peak temperatures and dimensions for DED 316L stainless steel cubic samples, as determined from thermal imaging contours. Values for the average maximum temperature and average melt pool area at the 5th, 15th, 25th, and 35th layers are presented for: (**a**) non-UV-A, and (**b**) UV-A conditions. The arrows indicate which axis each dataset corresponds to.

The thermal imaging camera was also used to measure the melt pool temperature profile while depositing the DED cubic samples. Results derived from these images for both non-UV-A and UV-A DED conditions are shown in Fig. 3. 800 frames were collected at layers 5, 15, 25, and 35 for both non-UV-A and UV-A conditions to estimate how the average peak melt pool temperature and average melt pool surface area evolve with increasing build size. The non-UV-A average maximum temperature and average melt pool area consistently increase until they reach an equilibrium state by layer 25. Under the UV-A condition, the average maximum temperature and average melt pool area values are larger at layer 5 but reach a steady state earlier, by layer 15. Statistical analysis shows that the differences in average maximum melt pool temperature and average melt pool surface area were statistically significant between the non-UV-A and UV-A conditions (p < 1.0 × 10^–15^ for most cases).

### High-speed video results

**Figure 4:**
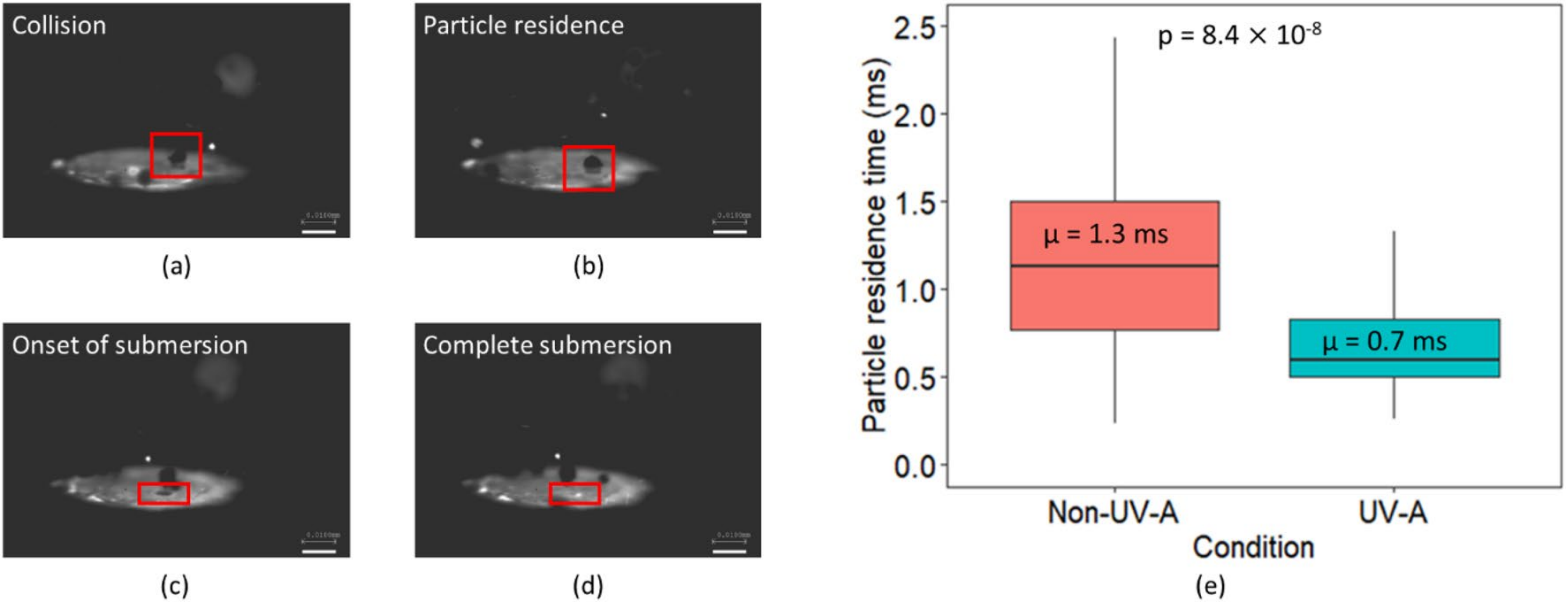
Representative images of (**a**) particle collision with melt pool surface, (**b**) particle residence on melt pool surface, (**c**) onset of particle submersion into melt pool, (**d**) particle submersion into the melt pool; and (**e**) particle residence time on the melt pool surface, measured using high-speed video imaging, for non-UV-A and UV-A conditions. The difference in particle residence time between the two conditions is statistically significant (p < 1.0 × 10^–7^). Video footage showing further details is available in the supplementary information.

High-speed video imaging was also used to measure particle residence time on the melt pool surface while depositing the single tracks, both for non-UV-A and UV-A conditions. Representative snapshots from particle collision with the melt pool surface to submersion into the melt pool, and the results for average particle residence time are shown in Fig. 4. Shown in Fig. 4 are representative snapshots of particle collisions with the melt pool surface (Fig. 4a), particle residence on the melt pool surface (Fig. 4b), the onset of particle submersion into the melt pool (Fig. 4c), and complete particle submersion into the melt pool (Fig. 4d). Acquired video footage illustrated in Fig. 4 is available in the supplementary information. The values for the average particle residence time, calculated from 100 particles for both non-UV-A and UV-A depositions, were 1.3 ± 0.32 ms and 0.7 ± 0.8 ms for the non-UV-A and UV-A deposits, respectively. The difference in particle residence time was shown to be statistically significant between the two conditions (p < 1.0 × 10^–7^), demonstrating that UV can effectively decrease particle residence time during DED, suggesting that the melt pool’s particle capture rate increases.

### Post-deposition microstructural characterization

Microscopy techniques, including optical microscopy, EBSD and SEM, were used to analyze non-UV-A and UV-A DED cubic samples to understand how UV influences as-deposited microstructures and defects, such as melt pool depth, grain size and porosity. Melt pool depth was measured by optical microscopy near the substrate, at the center of the sample, and near the top of cross-sectioned non-UV-A and UV-A cubes. Measured values for melt pool depth are summarized in Table 1. The difference in melt pool depth between non-UV-A and UV-A conditions for regions near the substrate was shown to be statistically significant (p < 1.0 × 10^3^). However, the difference in melt pool depth near the center and top was not statistically significant between the two conditions (p > 0.5).

**Table 1:** Comparison of non-UV-A and UV-A melt pool depth for the as-deposited 316L stainless steel cubic samples.

Location	Non-UV-A Melt pool depth (µm)	UV-A Melt pool depth (µm)	p-value
Near substrate	190 ± 16	235 ± 16	4.1 × 10^–4^
Center	215 ± 35	215 ± 28	0.96
Top	220 ± 33	200 ± 33	0.38

**Figure 5:**
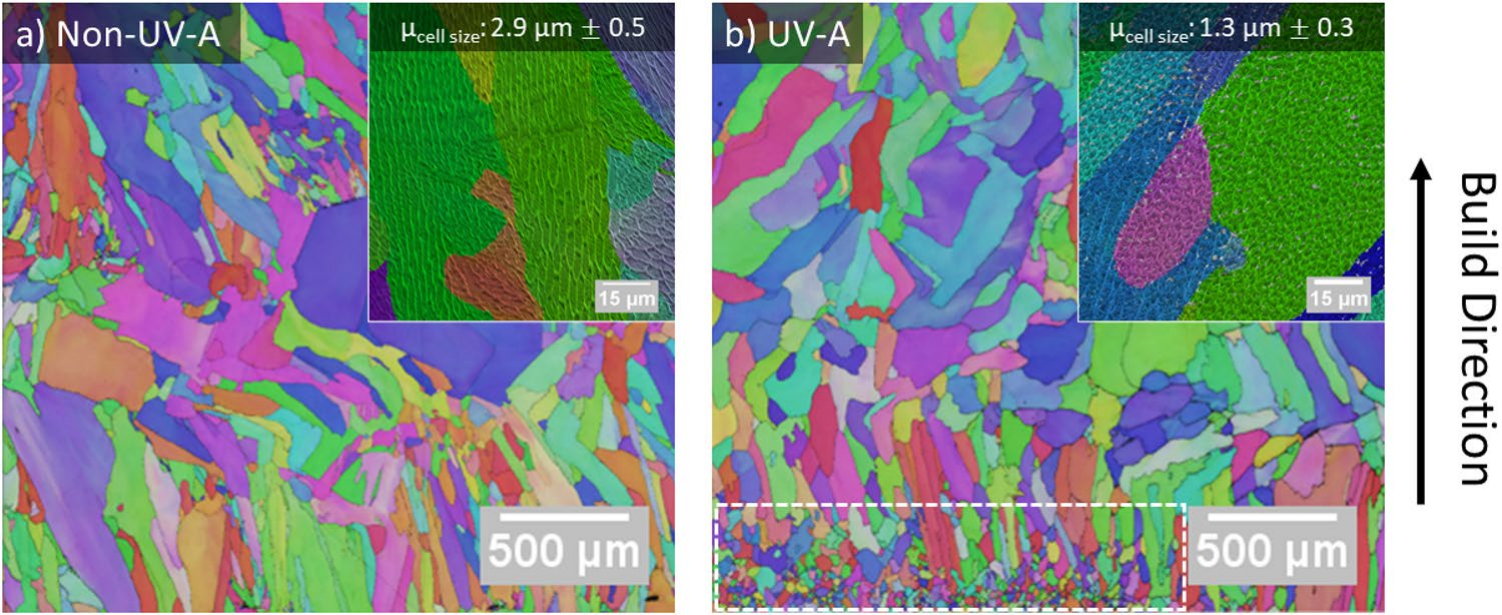
Microstructure analysis: Representative EBSD inverse pole figures of the as-deposited 316L stainless steel cubic samples near the substrate, for: (**a**) a non-UV-A sample, and (**b**) a UV-A sample. The insets show cellular dendrites in the non-UV-A and UV-A samples, respectively. The white, dashed box in (**b**) highlights the fine, equiaxed grains located in the first few layers of deposition.

Figure 5 shows EBSD analysis of microstructures of the cubes in the region near the substrate. As shown in Fig. 5a, the grains in the non-UV-A sample are columnar and tilted in the direction that the melt pool travels during deposition. Figure 5b shows finer, equiaxed grains located near the substrate, and a transition to a larger, columnar structure in the UV-A sample. SEM was used to collect images of cellular dendritic structures, and 100 data points per non-UV-A and UV-A conditions were used for statistical analysis. The insets in Fig. 5 show that the cellular dendrites are larger in the non-UV-A DED cubic samples than in the UV-A DED cubic samples, with average arm spacings of 2.9 ± 0.5 μm and 1.3 ± 0.3 μm, respectively. Statistical analysis indicates that the difference in average dendritic arm spacing between the non-UV-A and UV-A conditions was statistically significant (p < 1.0 × 10^–15^).

SEM was used to view and quantify the porosity in cubic samples. For statistical analysis of porosity, 50 data points were collected per non-UV-A and UV-A conditions. The non-UV-A DED cubes demonstrated large, distinct lack-of-fusion pores and small, scattered gas pores. The average percentage of porosity for the non-UV-A condition was 0.24% ± 0.35%. The UV-A DED cube displayed smaller, more scattered gas pores. Fine pores, on the order of ~ 150 nm in diameter, were observed at higher magnifications in the UV-A DED cube. These fine pores were concentrated at grain and cellular dendrite boundaries and were not observed in the non-UV-A samples. The average percentage of porosity for the UV-A condition was 0.02% ± 0.02%. The difference in the average percentage of porosity between the two conditions was statistically significant (p < 1.0 × 10^–4^).

## Discussion

### UV-induced phenomena within the melt pool

**Figure 6:**
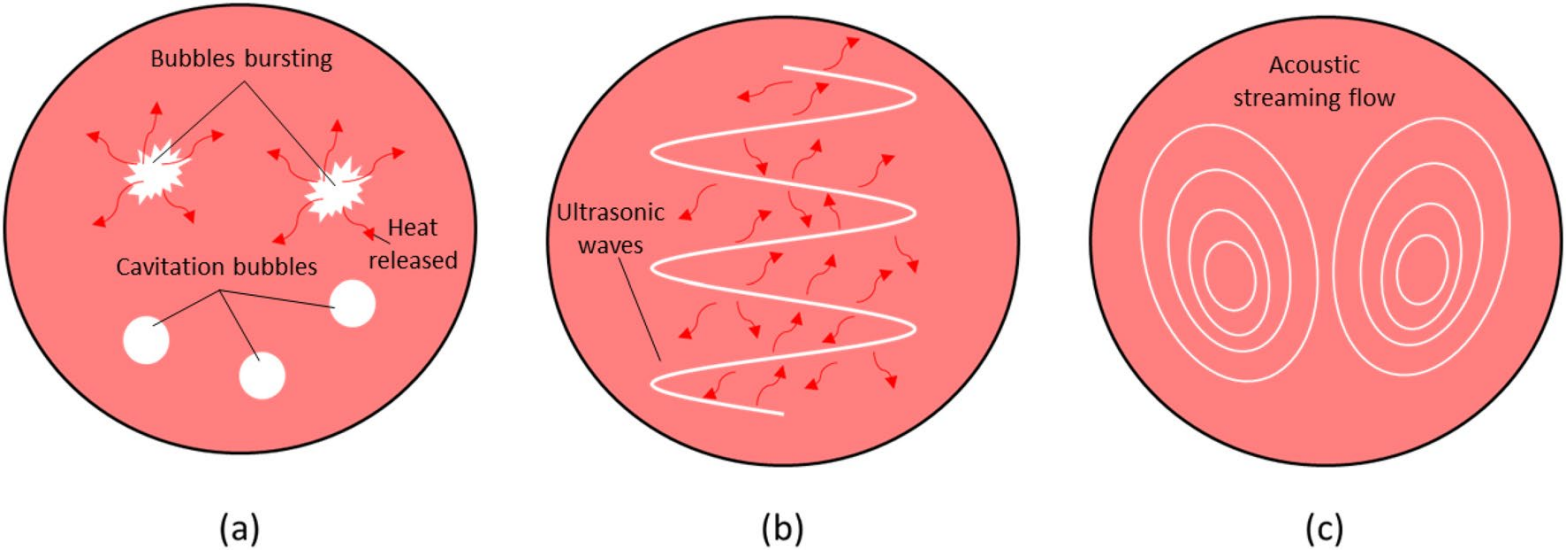
Schematic representations of the three UV-induced phenomena: (**a**) acoustic cavitation (i.e., the formation and collapse of cavitation bubbles that release heat into the melt pool), (**b**) ultrasound absorption (i.e., the dissipation of vibrational energy as heat into the melt pool), and (**c**) acoustic streaming (i.e., steady flow in the melt pool).

In UV-A treatment of molten metal, three phenomena have been reported to occur (Fig. 6), which are proposed to contribute to the distribution of excess heat within the melt pool observed in the present investigation (see Fig. 2). The first is acoustic cavitation, which is the rapid formation and collapse of gas voids^40^. As vibrational waves propagate through the melt, they form regions of high and low pressure. In the low-pressure regions, voids form and fill with gaseous species saturated in the surrounding melt. The bubbles then collapse. During the collapsing process, the liquid–gas interface implodes and builds inwards inertia, increasing the pressure and temperature within the bubble. Once the bubble collapses, the gaseous species are violently reincorporated into the surrounding liquid, carrying the generated heat with them. The second phenomenon is ultrasound absorption, which occurs as waves impart energy onto the molecules in the fluid as they pass through^41^. The transfer of energy results in deformation of the fluid during periodic compression and rarefaction. Viscous dissipation occurs, resulting in the transformation of kinetic energy into heat, which is then transmitted through the melt pool, raising its temperature^42^. The last phenomenon is acoustic streaming^9,43^. As a fluid interacts with acoustic oscillations, a transfer of momentum to the fluid occurs, resulting in steady fluid flow. The resulting fluid flow carries the higher-temperature liquid from the center of the melt pool to its outer edges, thereby improving heat transfer and stabilizing the temperature distribution in the melt pool. The three combined phenomena consequentially result in the observed increase in melt pool temperature and dimensions, as observed in Figs. 2 and 3.

We propose that combined acoustic cavitation, ultrasound absorption, and acoustic streaming have a direct impact on melt pool evolution during deposition, as observed from the *in situ* single-track results presented in Fig. 2 and from the cubic sample results for layer 5 presented in Fig. 3. The generation and transmission of heat from acoustic cavitation and ultrasound absorption contribute to the higher overall melt pool temperature of UV-A samples (Figs. 2 and 3). Furthermore, acoustic streaming and Marangoni flow drive the hotter regions of the molten metal towards areas of high surface tension (in this case, the melt pool boundaries)^44^. Thus, the solid liquid boundaries grow, and the melt pool dimensions increase (see Fig. 2).

### Effect of UV on interactions between powder and melt pool

The effects of UV on the melt pool temperature (see Figs. 2 and 3) influence the particle interactions with the melt pool surface. The particle residence time decreased by approximately 50% with UV, as shown in Fig. 4. The temperature-dependence of a fluid’s surface tension is expressed by the Eӧtvӧs rule^45^:

1 $${\gamma }_{lv}{V}^\frac{2}{3}=k({T}_{C}-T)$$

where *γ*_*lv*_ is the melt pool surface tension, *V* is the molar volume of the melt, *k* is a constant, *T*_*C*_ is the critical temperature at which surface tension is 0, and *T* is the melt pool temperature. As the melt pool temperature increases, *γ*_*lv*_ decreases. The decrease in *γ*_*lv*_ impacts the contact angle, as demonstrated by Young’s equation^46^:

2 $$\mathrm{cos}\theta =\frac{{\gamma }_{sv}-{\gamma }_{sl}}{{\gamma }_{lv}}$$

where *θ* is the contact angle between the particle and the melt pool surface, *γ*_*sv*_ is the particle surface energy, and *γ*_*s*l_ is the interfacial energy between the particle and the melt pool surface. As the *γ*_*lv*_ decreases, *θ* decreases. Additionally, due to the increase in melt pool temperature, *γ*_*sl*_ decreases, which also contributes to a decrease in *θ*^47^. Smaller values of *θ* indicate improved wettability of injected particles with the melt pool surface. Previous modeling by Haley et al. has shown that the particle residence time is shorter at smaller values of *θ*. Haley et al. also derived the temperature dependence of the particle residence time^48^:

3 $${t}_{melt}=\frac{1}{4\pi \alpha }{\left(\frac{8}{3}\pi {{r}_{p}}^{3}\frac{{T}_{0, pool}-{T}_{0, part}}{{T}_{0, pool}-{T}_{m}}\right)}^{2/3}$$

where *t*_*melt*_ is particle residence time, *α* is the thermal diffusivity, *r*_*p*_ is the particle radius, *T*_*0,pool*_ and *T*_*0,part*_ are the initial temperatures of the melt pool and particle respectively, and *T*_*m*_ is the melting temperature of the metal. According to the relationship in Eq. (3), as the melt pool temperature increases, the particle residence time decreases. This is consistent with present findings, shown in Figs. 2 and 4.

Historically, users have implemented a variety of techniques to improve powder particle wettability during DED processes, such as the addition of surface active elements^49–51^, powder surface treatments^52^, and powder coatings^53^. In this work, UV is shown to improve the wettability of injected particles with the melt pool surface without prior powder treatment, as indicated by the decrease in particle residence time noted in Fig. 4b. Ultimately, UV-A DED may allow users to bypass such techniques to improve wettability.

### Ultrasonic wave attenuation with increasing wave propagation distance

In standard non-UV-A DED, the substrate onto which parts are deposited, acts as a heat sink, leading to higher cooling rates within the bottom layers nearest the substrate. At higher layers, the substrate’s heat sink effects dissipate and heat accumulates, causing the melt pool to become hotter and larger^54^. The melt pool temperature and dimensions eventually reach a steady state and remain constant with increasing build height^55^. In the UV-A DED cubes, the melt pool reaches this steady state earlier than in the non-UV-A cubes, as indicated by the changes in melt pool depth (see Table 1) as well as average maximum melt pool temperature and average melt pool area (see Fig. 3). This observed behavior suggests that there is a threshold height by which, for the current configuration, the UV intensity is no longer large enough to activate the key phenomena discussed above that impart thermal energy to the melt pool. By the 25th and 35th layers, the heating effects caused by UV are negligible compared to the heat accumulation that is intrinsic to DED, as shown by the steady-state values for maximum average temperature (Fig. 3) and average melt pool surface area (Fig. 3), which are comparable to those for the non-UV-A condition.

In UV-A DED, ultrasonic waves are imparted through the melt pool, as well as the surrounding solid and mushy zones. As resonance conditions change, vibrational amplitudes vary with build height *z*. It is well documented that acoustic waves experience exponential attenuation as they travel through a medium. In polycrystalline solids, this attenuation can occur through several routes. One is ultrasound scattering, where waves are split at the interface between grains or different phases and are either transmitted or reflected. As this occurs, the waves attenuate accordingly^56^. The amount of scattering tends to increase in larger, anisotropic grains^57^. Ultrasonic waves can also attenuate in solids through ultrasound absorption. Similar to ultrasound absorption in fluids (i.e., the melt pool), ultrasonic energy is converted to heat and transmitted throughout the solid^57^. However, absorption in solids occurs through several different mechanisms, such as oscillation of dislocations, thermoelastic effects, and phonon or electron scattering^58^. As the build height increases, the attenuation of ultrasonic waves through scattering and absorption in the build likely increases, due to the presence of more grain boundaries and a larger bulk volume. Ultrasonic waves are also likely scattered at the melt pool boundaries.

The exponential attenuation of acoustic waves can be expressed by the following equation for acoustic intensity^1^:

4 $$I={I}_{0}{e}^{-2\alpha x}$$

where *I* is the ultrasonic wave intensity, *I*_*0*_ is the ultrasonic wave intensity at *z* = 0, *α* is the attenuation factor, and *x* is the wave propagation distance (in this case, *z* plus the thickness of the substrate). Assuming an attenuation factor of 0.2 for 316L stainless steel^59^, by the 15th layer of deposition, the UV intensity is approximately two-thirds of what it was at the first layer, and continues to decrease as *z* increases. However, the attenuation factor used in the previous calculation was measured at room temperature. As Wu et al. reports, ultrasonic waves experience greater amounts of attenuation at higher temperatures^60^. As the solid surrounding the melt pool experiences high temperatures, and due to thermal accumulation at higher layers, the decrease in intensity is likely greater than what is predicted by Eq. 4. Since a wave’s intensity is proportional to the energy it carries, less energy is transferred into the melt pool during the deposition of higher layers. Therefore, the effects of acoustic cavitation, ultrasound absorption in the fluid, and acoustic streaming weaken accordingly, leading to an overall reduction of heat transfer into the melt pool.

This presents an interesting challenge that must be addressed going forward in the development of UV-A DED. To maintain a constant ultrasonic wave amplitude with increasing build height, one of two solutions should be considered. The first is to tune the ultrasonic frequency as the build height increases, which can be accomplished with magnetostrictive transducers^61^. The second is to develop a top-down approach to applying vibration to the melt pool, so that the wave propagation distance remains constant during deposition. Additionally, the size and geometry of components should be strongly considered, as large geometries or complex shapes will influence how ultrasonic waves propagate through the build and into the melt pool.

### As-built microstructures and defects

The effects of UV on the melt pool’s evolution and its interactions with injected particles translate to the final build’s properties. Specifically, UV has an influence on solidification during deposition, as evidenced by the as-built microstructures. As shown in Fig. 5, fine equiaxed grains form near the substrate due to dendritic fragmentation. The pressure released from the collapse of cavitation bubbles mechanically fragments dendrites. Additionally, acoustic streaming transfers hot liquid to the dendrites, re-melting them. In this state, the steady flow exerts stress on dendrites and damages them^62–64^. The dendrite fragments thereby act as nucleation sites for fine grains.

A stronger effect of UV was observed on the cellular dendrites. As shown in the insets in Fig. 5, a decrease in dendritic arm spacing of nearly 50% occurred near the substrate. It is well documented that dendritic arm spacing decreases with increasing cooling rate^65,66^. As the cooling rate increases, there is insufficient time for lateral diffusion of rejected solute, thereby suppressing constitutional supercooling and leading to a smaller dendritic arm spacing^67^. It has been reported in casting and welding literature that vibration of molten metal causes a decrease in dendritic arm spacing^68–71^. The combination of acoustic streaming and collapse of cavitation bubbles enhance mixing of fluid, improving heat convection within the melt pool. Additionally, the melt pool growth results in a larger surface area for heat to conduct out of the melt. The enhanced heat transfer out of the melt pool through conduction and convection increases the cooling rate, thereby leading to the decrease in the dendritic arm spacing observed in Fig. 5.

Inducing UV within the melt pool is effective for mitigating harmful defects like lack-of-fusion porosity. Lack-of-fusion pores are large, irregularly shaped voids that form when successive melt pool layers fail to overlap one another^72^. The failure to overlap can be due to the improper selection of deposition parameters such as laser power, scan speed, spot size, and hatch spacing^73^; or partial melting of powder particles due to a lack of sufficient laser power for a given layer thickness and hatch spacing^15,74^. According to Mukherjee and DebRoy^73^, 316L stainless steel is particularly susceptible to the formation of lack-of-fusion porosity due to its high density, which results in smaller melt pools. To overcome the formation of such defects, it is recommended to optimize deposition parameters to achieve larger melt pools, a larger heat input into the melt pool, a higher Marangoni number, and a higher peak temperature within the melt pool^73^. Because of the three phenomena shown in Fig. 6, the melt pools of UV-A samples become larger and hotter, as observed in Figs. 2 and 3, leading to a lower average percentage of porosity. Our results indicate that UV-A DED can be used to achieve all four melt pool modifications suggested by Mukherjee and DebRoy^73^ without changing deposition parameters, thereby suppressing the formation of such defects. Typically, efforts to mitigate keyholing and lack-of-fusion pores are opposite^65^. UV-A DED provides a unique opportunity to address both defects within the same processing window.

However, UV does not necessarily eliminate all defects from builds. Miniscule pores on the order of ~ 150 nm in diameter were concentrated at the grain boundaries of the UV-A samples. Pores of such a small scale were not present in non-UV-A cubes. These small pores are likely residual cavitation bubbles that were not able to burst before being trapped at grain boundaries during solidification.

## Conclusions

The effects of UV on melt pool evolution in DED of 316L stainless steel single-tracks and cubes were explored. For the first time, in situ high-speed imaging was utilized to analyze particle collisions with the melt pool surface for UV-A and non-UV-A single-track depositions. In situ thermal imaging was also used to track the melt pool thermal profiles during UV-A and non-UV-A deposition of higher layers in bulk cubic samples. The use of in situ imaging techniques allowed for the collection of robust quantitative data, which were key to elucidating the effects of UV on the thermal and spatial evolution of the melt pool during UV-A DED. Using optical microscopy, SEM, and EBSD, the microstructures and porosity in the as-built bulk samples were extensively characterized. The specific findings of this investigation are as follows:

1. An increase in melt pool temperature and dimensions were observed during UV-A DED due to the combined effects of acoustic cavitation, ultrasound absorption, and acoustic streaming.
2. The particle residence time on the melt pool surface was reduced by approximately half, and wettability of injected particles into the melt pool surface improved, due to a decrease in surface tension caused by UV.
3. The cooling rate increases and dendritic arm spacing decreases during UV-A DED because of enhanced mixing in the melt pool caused by acoustic streaming.
4. Fewer lack-of-fusion pores were observed in UV-A cube samples because of an increase in melt pool dimensions and temperature. However, residual nanoscale acoustic cavitation bubbles are trapped as gas pores at grain boundaries during solidification.
5. An equiaxed-to-columnar grain transition is observed in UV-A cube samples because effects of UV on the melt pool diminish as the build height increases due to the attenuation of ultrasonic waves over an increasing wave propagation distance.

Overall, the findings indicate that the application of UV impacts the melt pool such that it solidifies with refined microstructures and fewer defects during DED processing. However, the UV effects diminish with build height. In the future, these limitations must be considered, as large or complex geometries may reduce the propagation of ultrasonic waves into the melt pool. A means of keeping the wave amplitude constant should be considered, either by tuning the wave frequency in situ, or by applying UV with a top-down approach.

## Methods

### Sample fabrication & in situ experimentation

Samples were deposited in an Optomec LENS® 750 workstation (Optomec, Albuquerque, NM, USA) with and without UV. Gas atomized 316L stainless steel (SLM Solutions, Lübeck, Germany) was used as feedstock powder. The particle size distribution was between 25 and 150 µm. The chemical composition of the powder, as provided by the manufacturer, is shown below in Table 2.

**Table 2:** Chemical composition (weight %) of 316L stainless steel feedstock powder, as provided by the manufacturer.

Fe	Cr	Ni	Mo	Mn	Si	P	S	C	N
Balance	16.00–18.00	10.00–14.00	2.00–3.00	2.00	1.00	0.045	0.030	0.030	0.10

The samples were deposited onto a 316L stainless steel substrate (McMaster Carr, Elmhurst, IL, USA), with a length and width of 150 mm, and a thickness of 6 mm. A piezoelectric ultrasonic transducer (Beijing Ultrasonic, Beijing, China) was coupled to the bottom-center of the substrate using a high-temperature resistant epoxy (JB Weld, Sulphur Springs, TX, USA). A dampener (Grainger, Los Angeles, CA, USA) was placed beneath the transducer to prevent damage to the build plate. The assembly was fastened to the LENS® build plate. The transducer was set to an ultrasonic frequency of 33 kHz and an ultrasonic amplitude of 5 µm.

Single tracks were created under an ambient atmosphere. Single tracks were 10 mm long and 1 mm wide. These experiments were recorded with a FASTCAM SA-Z 2100 K M4 high-speed video camera (Photron USA, Inc., San Diego, CA, USA) at a frame rate of 30,000 frames per second. Cubic samples (40-layer, 10 mm × 10 mm) were deposited under an inert argon atmosphere with oxygen maintained at < 100 ppm. A charged couple device (CCD) thermal imaging camera was used to capture melt pool thermal profiles at 500 frames per second, from a top-down view of the x–y plane, for all depositions. For the cubes, thermal imaging data were captured every 10 layers, starting at the 5th layer. All samples were deposited with a laser power of 390 W, a layer thickness of 0.2 mm, a scan speed of 16.9 mm/s, a hatch spacing of 0.5 mm, and a volumetric energy density (VED) of 228 J/mm^3^. The beam diameter is estimated to be 0.84 mm based on previous studies using the same instrumental setup^75^. Volumetric energy density (VED), *E*_*v*_*,* was calculated with the following equation^76^:

5 $${E}_{v}=\frac{P}{v\cdot t\cdot h}$$

where *P* is laser power, *v* is laser scan speed, *t* is layer thickness, and *h* is hatch spacing. The powder feed rate was 22 g/min for single tracks, and 27.5 g/min for cubic samples.

### Analysis of in situ results

High-speed video camera footage of single track depositions was viewed and analyzed with the Photron FASTCAM Viewer 4 software^77^. Analysis of 100 particles that collided and submerged near the center of the melt pool was done for both non-UV-A and UV-A conditions. Particle residence time was calculated by measuring the amount of time between a particle’s collision with the melt pool surface, and its submersion into the melt pool.

The thermal imaging camera data for the single track and cube depositions were analyzed using Python^78^. A script was written to conduct statistical analysis on the melt pools’ average maximum temperature and surface area. Thermal contour maps and profiles of melt pool surfaces were generated accordingly. Error bars were calculated from standard deviation values from approximately 1000 frames at each layer.

### Defect and microstructural characterization

The as-built 316L stainless steel cubes were removed from the substrate and cross-sectioned with a FA20S electrical discharge machine (Mitsubishi, Tokyo, Japan). The samples were hot-mounted in a KonductoMet® conductive mounting compound (Buehler, Lake Bluff, IL, USA), and polished to a 0.05 μm finish using a RotoPol-22 polisher (Struers, Copenhagen, Denmark).

SEM of porosity and microstructures was performed using a field emission (FE) Magellan 400 XHR microscope (FEI, Hillsboro, OR, USA) with an accelerating voltage of 20 kV. Quantitative analysis of images was done using ImageJ software^79^. Color thresholding was used on porosity to calculate the total percent of the image area attributed to porosity. EBSD was carried out in the FE-SEM equipped with EBSD-symmetry detectors (Oxford Instruments, Abingdon, United Kingdom), with a step size of 1 μm. Oxford AZtec software was used for EBSD data processing and microstructural analysis^80^.

The cross-sectioned cubic samples were etched in a solution of HCl and HNO_3_ in a 3:1 ratio for 30 s. They were immediately rinsed in a cold-water bath to prevent the etchant from reacting further with the sample surfaces. Images of melt pool boundaries were taken with a BX53M optical microscope (Olympus, Tokyo, Japan) at the bottom (nearest the substrate), center, and top of the samples. Melt pool boundaries were traced digitally. Cellular dendritic structures were imaged with SEM. Using ImageJ, melt pool depth and dendritic arm spacing were measured from the optical and SEM micrographs, respectively. Dendritic arm spacing was measured with an area counting method^81^. Statistical analysis was done on 100 measurements per non-UV-A and UV-A condition. Two-sample t-tests were done in the software R^82^.

### Supplementary Information

Supplementary Video 1.

Supplementary Information 1.

## Data Availability

All data used to support the conclusions in the paper are present in the paper and/or supplementary information. Additional data is available from the corresponding author upon request.
