# Supplementary material for: In situ observation of melt pool evolution in ultrasonic vibration-assisted directed energy deposition
Source: Sci Rep. 2023 Oct 17;13:17705. doi: 10.1038/s41598-023-44108-4 (PMC10582076; doi:10.1038/s41598-023-44108-4)
Supplement: Supplementary file 2 — Supplementary Information 1. [file 41598_2023_44108_MOESM2_ESM.docx]

Supplementary video S1:

High-speed video clip of particle collision with melt pool surface and submersion into the melt pool
